# Regulation of the High Affinity IgE Receptor (FcεRI) in Human Neutrophils: Role of Seasonal Allergen Exposure and Th-2 Cytokines

**DOI:** 10.1371/journal.pone.0001921

**Published:** 2008-04-02

**Authors:** Martin P. Alphonse, Arash S. Saffar, Lianyu Shan, Kent T. HayGlass, F. Estelle R. Simons, Abdelilah S. Gounni

**Affiliations:** 1 Department of Immunology, Faculty of Medicine, University of Manitoba, Winnipeg, Manitoba, Canada; 2 Department of Pediatrics and Child Health, Faculty of Medicine, University of Manitoba, Winnipeg, Manitoba, Canada; Albert Einstein College of Medicine, United States of America

## Abstract

The high affinity IgE receptor, FcεRI, plays a key role in the immunological pathways involved in allergic asthma. Previously we have demonstrated that human neutrophils isolated from allergic asthmatics express a functional FcεRI, and therefore it was of importance to examine the factors regulating its expression. In this study, we found that neutrophils from allergic asthmatics showed increased expression of FcεRI-α chain surface protein, total protein and mRNA compared with those from allergic non asthmatics and healthy donors (p<0.001). Interestingly, in neutrophils isolated from allergic asthmatics, FcεRI-α chain surface protein and mRNA expression were significantly greater during the pollen season than outside the pollen season (n = 9, P = 0.001), an effect which was not observed either in the allergic non asthmatic group or the healthy donors (p>0.05). Allergen exposure did not affect other surface markers of neutrophils such as CD16/FcγRIII or IL-17R. In contrast to stimulation with IgE, neutrophils incubated with TH2 cytokines IL-9, GM-CSF, and IL-4, showed enhanced FcεRI-α chain surface expression. In conclusion, these results suggest that enhanced FcεRI expression in human neutrophils from allergic asthmatics during the pollen season can make them more susceptible to the biological effects of IgE, providing a possible new mechanism by which neutrophils contribute to allergic asthma.

## Introduction

Asthma is a chronic inflammatory disease of the bronchial airways which has been increasing in prevalence during the last four decades. Airway inflammation is a major factor in the pathogenesis of asthma, in associated bronchial hyperresponsiveness and in disease severity [1], [2]. The inflammatory component of this disease includes an increased number of activated T lymphocytes, mast cells, neutrophils and eosinophils within the airway lumen and bronchial submucosa [3].

Many studies support the concept that neutrophils may significantly contribute to chronic inflammation and alterations in airway structure that characterize asthma. After allergen challenge of patients with allergic asthma, neutrophils are the first inflammatory cells to accumulate within the airways and neutrophil numbers in bronchoalveolar lavage (BAL) fluid of patients with allergic asthma after allergen challenge have been calculated to be about 90 times higher than healthy controls [4], [5], [6]. Furthermore, an increase of airway neutrophils was also detected in induced sputum from adults with acute exacerbations of severe asthma, and in bronchial biopsies of severe steroid resistant asthmatics [7], [8], [9]. Circulating neutrophils are activated during active asthma, after exercise-induced bronchospasm and during both early and late asthmatic reactions induced by allergen [7].

IgE has long been regarded as a major molecular component of atopic diseases, including asthma [10]. Clinical studies have found a close association between allergic asthma and elevated serum specific IgE levels as well as IgE dependent skin test reactivity to allergens [11]. In addition, allergen specific IgE has been detected in bronchoalveolar lavage fluids in asthmatic patients [12].

The high affinity receptor for IgE (FcεRI) is a key structure involved in immediate allergic manifestations [13]. Initially discovered on mast cells and basophils whose function is to mediate cellular degranulation and the release of various mediators such as histamine [13], FcεRI has been later detected on many inflammatory cells including human cutaneous Langerhans cells, dendritic cells, monocytes of patients with a number of allergic disorders, on eosinophils from subjects with hypereosinophilic syndrome or asthma, on platelets, and bronchial epithelial cells [14], [15], [16], [17], [18].

Recently, we demonstrated that human neutrophils isolated from allergic asthmatics express FcεRI [19] and thus, it was of importance to examine the factors regulating expression of FcεRI in human neutrophils. Here, we show that human neutrophils from allergic asthmatics express high levels of FcεRI compared with allergic non asthmatics and healthy donors. Furthermore, natural seasonal allergen exposure up-regulates FcεRI expression at mRNA and cell surface levels in neutrophils of allergic asthmatics but not in allergic non asthmatics or healthy donors. This effect is selective since seasonal allergen exposure did not alter surface markers of neutrophil such as FcγRIII/CD16. Interestingly, *in vitro* stimulation with TH-2 cytokines including IL-9, GM-CSF and IL-4 induced elevated FcεRI expression in neutrophils from allergic asthmatics.

## Results

### Increased expression of FcεRI in human neutrophils from allergic asthmatics compared to non asthmatic allergic and healthy individuals

**Figure 1:**
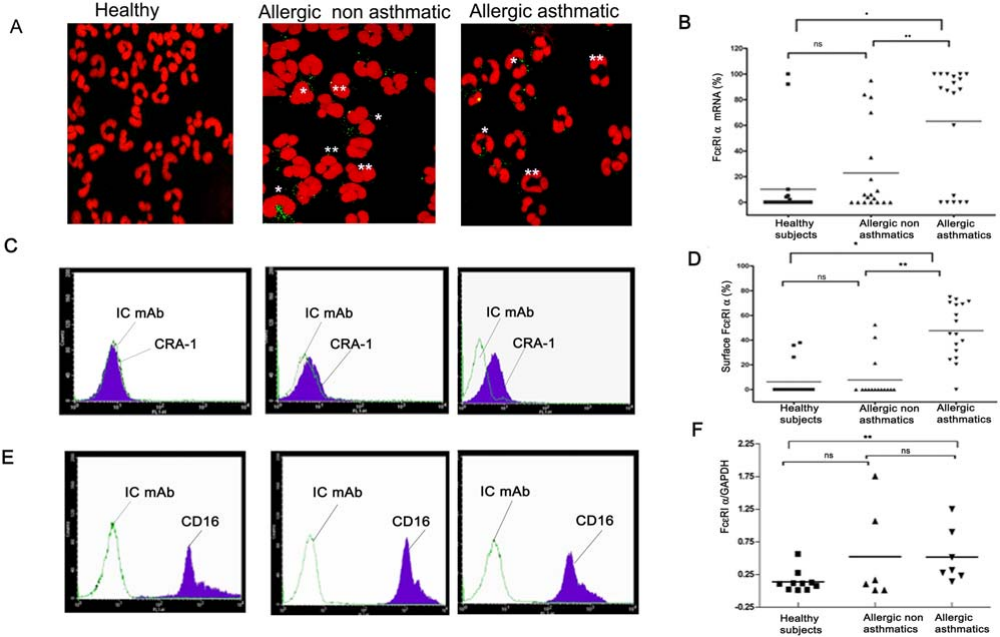
Upregulated FcεRI α chain mRNA and surface expression in neutrophils from allergic asthmatics. Neutrophils FcεRI α chain mRNA and surface expression in allergic asthmatics, allergic non asthmatics and healthy subjects were determined by FISH (A,B) real-time RT-PCR (F) and flow cytometry (C, D), respectively, as described in Methods. E. FcγRIII/CD16 surface expression on neutrophils of the same subjects in A and C. , *p<0.0001, **p<0.01, ns: non significant. P values were calculated using Mann Whitney U test. IC mAb: isotype control. Example of cells with positive (one asterisk) and negative (two asterisks) signal in FISH are indicated in panel A.

Expression of FcεRI by cells other than mast cells was previously reported, as was expression in neutrophils [19]. To investigate the level of FcεRI expression in human neutrophils from allergic asthmatic, allergic non asthmatic and healthy individuals, we first analyzed FcεRI mRNA expression by fluorescent *in situ* hybridization (FISH) using anti-sense FcεRI-α chain riboprobe. Neutrophils from allergic asthmatics showed an increase in FcεRI mRNA expression (mean percentage of positive cells 63.2±10.21%, n = 19) compared with allergic non asthmatic (22.9±8.08%, n = 18) or healthy individuals (10.14±6.25 %, n = 21) (p<0.001, Figure 1A, B). No specific signal could be detected with sense riboprobe used as negative control (data not shown). Furthermore, real-time RT-PCR analysis showed a significant increase of FcεRIα chain mRNA in neutrophils from allergic asthmatics (n = 7) compared with healthy individuals (n = 10, p = 0.0046), but not allergic non asthmatics (n = 6, p>0.05, Figure 1F). Moreover, neutrophils from allergic asthmatics express 1000 to 300 times less FcεRI-α mRNA compared to basophilic cell line KU812 (relative mean copy number in KU812 = 737±134/GAPDH copy, n = 3).

To confirm that neutrophils from allergic asthmatics expressed FcεRI at cell surface, flow cytometry analysis using mAb directed against the FcεRI α chain, the IgE binding subunit [20], was performed. Neutrophils from allergic asthmatics, which express the neutrophil marker CD16 (Figure 1E), showed an increase in FcεRI surface expression (% of positive cells 47.67±5.46%; Mean fluorescence intensity (MFI) = 49.5±12.37, n = 17) compared with neutrophils from allergic non asthmatics (7.74±4.41%; MFI = 3.54±2.3; n = 15) and healthy individuals (6.23±3.39%; MFI = 0.65±0.3; n = 16) (p<0.001, Figure 1C, D). Interestingly, we found that neutrophils express between 1.3 to 10 times less FcεRI surface receptor compared with basophilic cell line (KU812) by using quantitative indirect immunofluorescence (Supplemental Figure 1B). We further analyzed the same samples by immuno-fluorescence and found an increase in FcεRI protein expression in neutrophils from allergic asthmatics (% of positive cells: 86.92±9.03 %; n = 10) compared with allergic non asthmatics (28.82±13.47%; n = 11) and healthy donors (0.4±0.5; n = 11).

### Allergen exposure during pollen season up-regulates FcεRI expression by human neutrophils from allergic asthmatics

**Figure 2:**
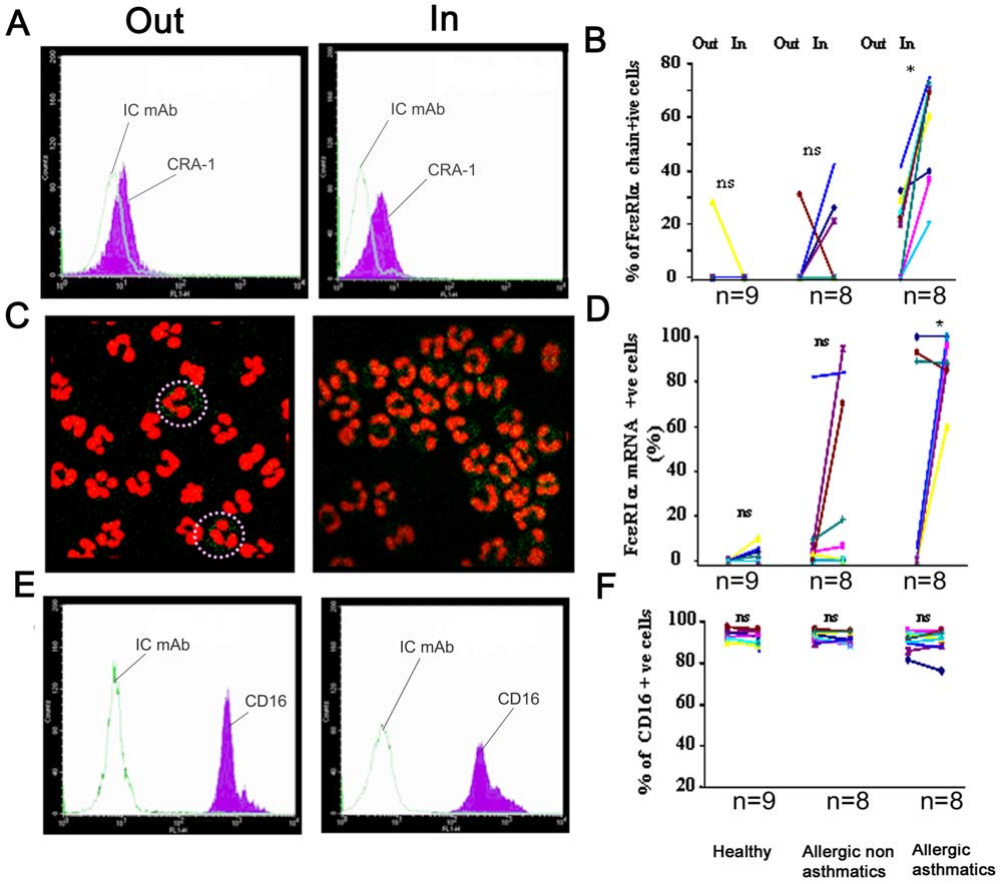
Effect of pollen season on surface and mRNA expression of FcεRI in human neutrophils. FcεRI α chain surface (A, B) and mRNA expression (C, D) are up-regulated during the pollen season in human neutrophils from allergic asthmatics, but not in allergic non asthmatics or healthy donors. *p<0.01, ns: non significant. *P* values were calculated using Wilcoxon signed rank test. (E, F) represent CD16 surface expression in neutrophils from the same subjects as in (A, B) and (C–D).

We then investigated whether allergen exposure during the pollen season can account for the increased expression of FcεRI in human neutrophils from allergic asthmatics. FcεRI expression was tested in and out of the pollen season in neutrophils from the same allergic asthmatics, non allergic asthmatics and healthy donors. Flow cytometry analysis of human neutrophils from allergic asthmatics showed an increase in FcεRI surface expression during the pollen season (57.37±18.7 during versus 18.8±14.5 out of season, n = 9, P<0.001, Figure 2A, B). FISH and immunofluorescence analysis revealed a similar pattern with an increase of FcεRI α chain mRNA positive neutrophils (Figure 2 C, D) and FcεRI-α chain immuno-positive neutrophils (data not shown) during the pollen season compared with neutrophils obtained out of the pollen season.

To confirm that seasonal allergen exposure selectively affected FcεRI expression in neutrophils from allergic asthmatics, surface expression of human neutrophil markers FcγRIII/CD16 and IL-17AR was investigated by FACS in neutrophils isolated from the same allergic asthmatic subjects. As detected by FACS analysis FcγRIII/CD16 (Figure 2E–F) and IL-17R (data not shown) expression was stable during and out of the pollen season (p>0.05). Furthermore, seasonal allergen exposure did not affect FcεRI surface or mRNA expression by human neutrophils from allergic non asthmatics and healthy individuals (p>0.05, Figure 2B and D).

### Effect of TH-2 cytokines on FcεRI expression in neutrophils from allergic asthmatics

**Figure 3:**
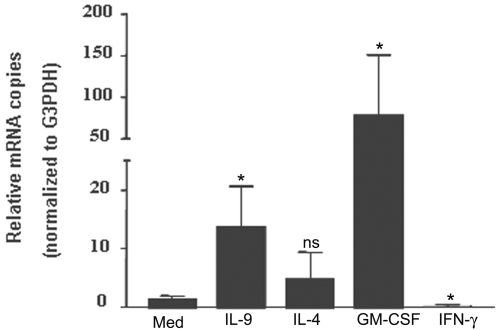
TH-2 cytokines up-regulate FcεRI α mRNA expression in human neutrophils isolated from allergic asthmatics. Neutrophils from three different asthmatics stimulated with IL-9, IL-4, or GM-CSF (10ng/ml) for 6h showed increased mRNA expression of FcεRI α chain as determined by Real-time RT-PCR. Data represents the mean±SEM of three independent experiments performed under the same condition. * P<0.05 compared with medium alone and were calculated using Mann Whitney U test.

**Figure 4:**
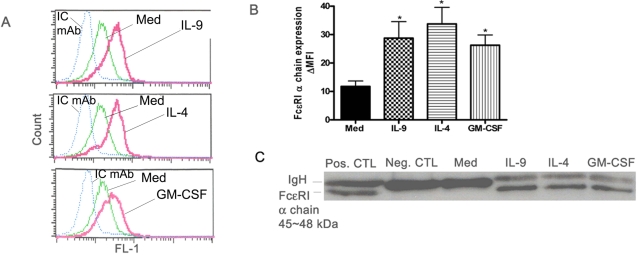
TH-2 cytokines induce FcεRI α chain surface expression in neutrophils from allergic asthmatics. A. Representative FACS data of FcεRI α chain surface expression in neutrophil stimulated with IL-9, IL-4, or GM-CSF (all at 10ng/ml) for 18hrs. FACS was performed using mAb anti-FcεRI α chain (CRA-1). B. Each point represents mean±SEM values from four allergic asthmatic donors. Results are expressed as MFI of CRA-1 minus MFI of isotype control mAb (ΔMFI). * P<0.05 compared with with medium alone and were calculated using Mann Whitney U test. (C) Total protein lysates were subjected to immunoprecipitation with IgE/anti IgE and Western blotting with CRA-1 as described in Methods. Basophilic cell line (KU812) was used as positive control. Negative control corresponds to neutrophil protein lysate analyzed without IgE/anti-IgE immunoprecipitation. Data in C is representative of three separate experiments.

To investigate the potential mechanism through which seasonal allergen exposure might influence FcεRI expression in human neutrophils from allergic asthmatics, we studied the effect of Th-2 cytokines on FcεRI expression *in vitro*. Expression of FcεRI mRNA and protein was analyzed following stimulation with IL-4, IL-9 or GM-CSF for 6hr. The number of FcεRI-α chain mRNA copies was consistently higher in IL-9 or GM-CSF stimulated cells than in unstimulated neutrophils, as measured by real-time RT-PCR in three separate experiments (Figure 3). Although IL-4 seems to enhance FcεRI α chain mRNA expression in human neutrophils it did not reach significance (p>0.05). Moreover, stimulation with IFN-γ decreased the expression of FcεRI mRNA in neutrophils from allergic asthmatics (n = 3, P<0.05, Figure 3). We then confirmed the effect of TH-2 cytokines on FcεRI expression at the surface and intracellular protein level. As detected by flow cytometry, IL-4, GM-CSF and IL-9 up-regulated significantly FcεRI α chain surface expression in human neutrophils from allergic asthmatic individuals (n = 4, p<0.05, Figure 4A and B). Furthermore, in contrast to medium treated cells, significant up-regulation of FcεRI-α chain 45∼48 kDa was detected in IL-9, IL-4 and GM-CSF stimulated neutrophils as detected by immunoprecipitation with IgE/anti IgE and Western blot using anti-FcεRI α chain mAb (Figure 4C, n = 3).

## Discussion

IgE has long been regarded as a major molecular component of allergic diseases, including asthma[10]. The effect of IgE is amplified by the activities of the receptors to which it binds. The high affinity receptor for IgE (FcεRI) is a key structure involved in immediate allergic manifestations. Recently, it has become apparent that neutrophils from allergic asthmatics express FcεRI [19]. Although this reveals the capability of these cells to respond to stimuli considered integral to the allergic process, factors that modulate FcεRI expression by human neutrophils have not been investigated. In our study, we have demonstrated that neutrophils from allergic asthmatics have increased FcεRI expression compared with healthy donors or allergic non asthmatics. Interestingly, seasonal allergen exposure selectively enhances the surface and mRNA expression of FcεRI in human neutrophils from allergic asthmatics and not in allergic non asthmatics or healthy donors. Furthermore, neutrophils from all groups showed no change in surface expression of CD16/FcγRIII and IL-17R during and out of pollen season. Moreover, TH-2 cytokines, highly expressed in human allergic asthma, mimicked seasonal pollen exposure *in* vitro and up-regulated FcεRI expression in neutrophils from allergic asthmatics (Figure 3 and 4) but not from allergic non asthmatics or healthy donors (data not shown). Taken together, our data provide a potential new mechanistic explanation of increased neutrophil presence and activity in allergic asthma [21].

For many years, FcεRI was thought to be exclusively expressed on mast cells and basophils which mediate cellular degranulation and the release of various mediators such as histamine, leukotrienes and a number of cytokines and chemokines, the hallmark of allergic reactions [13]. Later, FcεRI was also detected on human cutaneous Langerhans cells, monocytes from patients with allergic disorders, eosinophils from patients with hypereosinophilic syndrome or asthma, on platelets, circulating dendritic cells and bronchial epithelial cells[13], [18]. FcεRI expressed on monocytes and circulating dendritic cells was shown to mediate calcium flux [15], and to promote IgE mediated antigen presentation [16]. We assessed the expression of FcεRI in human neutrophils among the three groups of individuals in two ways. First, we determined the mRNA expression of FcεRI-α chain using *in situ* hybridization. Second, we compared surface and total protein expression of FcεRI-α chain using flow cytometry and immunofluorescence, respectively. These analyses showed that neutrophils in allergic asthmatics but not in allergic non asthmatics or healthy individuals expressed high levels of mRNA, surface and intracellular protein FcεRI. In accordance with our results, high levels of FcεRI expression have been observed in monocytes, eosinophils and Langerhans cell from allergic subjects compared with healthy donors [13]. Interestingly, heterogeneity in FcεRI expression is observed within neutrophils from allergic asthmatic at the level of both mRNA and protein, suggesting that FcεRI expression in neutrophils is highly regulated.

We then investigated whether seasonal exposure to allergen affects FcεRI in neutrophils from the three groups. Our data clearly demonstrated specific up-regulation of FcεRI at the mRNA and protein level in neutrophils isolated *ex vivo* during the pollen season (Figure 2). Expression of other surface receptors by human neutrophils was not affected, suggesting that FcεRI may play a role in allergic asthma during pollen season.

Studies that have investigated the regulation of FcεRI *in vivo* and *in vitro*, have shown a correlation between the serum IgE level and FcεRI expression on the cell surface [22], [23]. Two hypotheses have been proposed to explain the basis of this correlation. Either a common factor was capable of regulating the level of both serum IgE and IgE receptors, or the number of receptors was modulated by serum IgE concentrations [24], [25]. Subsequent studies have demonstrated that monomeric IgE up-regulates FcεRI expression on human mast cells and mouse basophils [23], [26], [27]. The proposed mechanism of this up regulation is that IgE protects the cell bound receptors from degradation during receptor recycling [13]. Mast cells from IgE deficient mice express low levels of FcεRI unless up-regulated by *in vitro* incubation of these cells with IgE or injection of IgE *in vivo* [28]. However, our data revealed that *in vitro* incubation with IgE has no effect on FcεRI surface expression in human neutrophils isolated from allergic asthmatic (Figure S1,A) or allergic non asthmatic individuals (data not shown). This phenomenon may occur through various plausible mechanisms. First, stabilization by IgE, which prevents FcεRI receptor internalization and degradation, may not have occurred in neutrophils, in contrast to human basophils [29], [30]. Second, the preformed pool of FcεRI receptor was possibly not adequately transported to the cell surface. Third, the basal level of FcεRI protein synthesis was not maintained in cultured neutrophils, due to their well documented low transcriptional and translational capacity, thus preventing intracellular pool and cell surface replenishment. Fourth, it may be that IgE prevents FcεRI surface loss within 24 hrs of culture but simultaneous apoptosis occurring in neutrophils with associating membrane changes may disguise this effect. Further in depth studies are needed to address these possibilities.

Regulation of FcεRI expression is not entirely dependent on IgE. Mast cells from IgE deficient mice express low levels of FcεRI [28] which suggests that the basal levels are under the control of other regulators. In this study, we have clearly demonstrated that Th-2 cytokines IL-9, GM-CSF and IL-4, upregulate FcεRI protein expression in human neutrophils. These data are in agreement with a positive role of IL-4 and IL-9 in the transcription of FcεRI-α chain in human mast cells, eosinophils from patients with atopic dermatitis and human dendritic cells [31], [32], [33]. Interestingly, although IL-4 did significantly increase FcεRI surface expression in human neutrophils it's effect at mRNA level was not consistent which may suggest that IL-4 affects FcεRI expression by a different mechanism than GM-CSF and IL-9. Furthermore, we also demonstrated that GM-CSF upregulates FcεRI in human neutrophils (Figure 3). As a key cytokine with pleotropic activity on neutrophil function, GM-CSF also has the ability to induce other neutrophil surface receptors, including the low affinity IgE receptor CD23/FcεRII [34]. Taken together, our data highlight the fact that Th-2 cytokines directly and indirectly account for the pathophysiologic manifestations of allergy and are involved in inducing FcεRI, hence amplifying the IgE/ IgE receptors network.

In previous studies, we and others have demonstrated that FcεRI dependent activation of neutrophils from allergic asthmatics leads to IL-8 release [19], [35], a potent neutrophilic chemoattractant, as well as proteases that may damage airway mucosa [35], [36], [37]. More recently, we have demonstrated that IgE binding to FcεRI can enhance neutrophil survival in allergic asthmatics by a mitochondrial dependent mechanism [21]. In light of these studies and our present data, we propose that, *in vivo*, allergen exposure in sensitized asthmatics not only enhances neutrophil recruitment to the site of allergen exposure but also upregulates expression of FcεRI by these cells which may therefore participate in IgE-mediated allergic inflammatory responses.

## Methods

### Subjects

This study was approved by the Ethics Committee of the Faculty of Medicine, University of Manitoba, Winnipeg, Canada and written informed consent was obtained from each participant. In response to advertisements, individuals 18–45 years old were recruited in each of three groups: allergic individuals with mild asthma, allergic non-asthmatics, and healthy donors. The clinical diagnosis of allergic asthma was determined by: (i) history of asthma symptoms (wheeze, cough, and/or shortness of breath) during the short (6–8 week long) local grass pollen season, controlled with albuterol as needed; (ii) positive epicutaneous test to mixed grass pollen (wheal diameter at least 3 mm more than histamine control wheal) to an epicutaneous test with mixed grass pollen; (iii) 15% or greater improvement in forced expiratory volume in one second (FEV_1_) after inhalation of albuterol (200 µg) from a metered-dose inhaler. The clinical designation of allergic non-asthmatic was determined by: (i) history of allergic rhinitis symptoms (sneezing, nasal itching, discharge, and/or congestion) during the short local grass pollen season, relieved by an H_1_-antihistamine as needed; (ii) positive epicutaneous test to mixed grass pollen (wheal diameter at least 3 mm more than histamine control wheal), (iii) no history of asthma symptoms at any time of year, normal FEV_1_ and no change in FEV_1_ after albuterol 200 µg from a metered-dose inhaler. The healthy donors had no history of asthma, allergic rhinitis, or other allergic disease, negative epicutaneous tests to mixed grass pollen, normal FEV_1_ and no change in FEV_1_ after albuterol 200 µg by metered-dose inhaler. Study participants had not received allergen-specific immunotherapy. For three days before collection of a 40 ml blood sample, all participants refrained from using all medications, including β_2_-adrenergic agonists and H_1_-antihistamines. Participants who reported an upper respiratory tract infection within the previous month were excluded from the study.

### Reagents and antibodies

FITC-conjugated AffiniPure rat anti-mouse IgG (H+L) and F(ab)′_2_ were obtained from Jackson ImmunoResearch Laboratories (West-Grove, Pennsylvania). Monomeric IgE was purchased from Diateck (Oslo, Norway). Murine anti-human IgE mAb was purchased from BD Biosciences Pharmingen (Mississauga, Ontario). Murine anti-FcεRIα chain mAb CRA-1 (mouse IgG1) which recognizes non IgE-binding site was kindly provided by Dr. Chisei Ra, Tokyo University, Japan. FITC labelled mouse anti-human CD16 mAb (Clone 3G8, IgG1), FITC labelled and unlabeled mouse IgG1 isotype control mAb, (clone MOPC21, IgG1) were obtained from Sigma Chemical Co. (Oakville, Ontario). Anti-CD16 mAb immunomagnetic beads were obtained from Miltenyi Biotec (Auburn, Calif). Recombinant human IL-9, IL-4, GM-CSF, and IFN-γ were purchased from R&D Systems MN). LightCycler-DNA Master SYBR Green I, digoxigenin UTP, and mouse monoclonal anti-digoxiginin were purchased from Roche (Mississauga, Ontario). Ficoll-Paque™ Plus and Dextran 500 were obtained from Amersham Pharmacia Biotech AB (Baie d'Urfé, Quebec). HyQ® RPMI 1640 and heat inactivated foetal bovine serum were obtained from Hyclone Laboratories (Logan, Utah). ProLong® antifade, propidium iodide, Tyramide Signal Amplification Kit were obtained from (Invitrogen, Carlsabd, CA). Unless stated otherwise, all other reagents were obtained from Sigma Chemical Co.

### Cell preparation and culture

Human neutrophils were purified from peripheral blood as we described previously [19]. Viability of the cells was >98% as assessed by trypan blue dye and purity ranged from 96 to 99%. Freshly isolated neutrophils (2×10^6^/ml) from three allergic asthmatic subjects were incubated at 37°C in humidified 5% CO_2_ in RPMI 1640 medium supplemented with 10% heat inactivated FBS and antibiotics for 6 or 18 hrs at 37°C with IL-9, IL-4, GM-CSF, or IFN-γ at a final concentration of 10ng/ml. After culture, cells were washed and either stained for FcεRI-α chain surface protein or subjected to RNA isolation and protein extraction for real time RT-PCR and Western blot analysis. Cytospin slides were prepared from freshly isolated or cytokine stimulated neutrophils as previously described [19].

### Flow cytometry

Flow cytometry analysis was performed by incubating freshly isolated or cytokine stimulated neutrophils (1×10^5^) cells for 30 min in PBS/5% FBS with mAb CRA1 or isotype control mAb IgG_2a_ (final concentration 10 µg/ml) [19]. The cells were washed twice with PBS/2%FBS and incubated for 30 min with affinity purified FITC conjugated rat anti-mouse IgG F(ab)′_2_ (1∶200). After washing, cells were fixed with 2% PAF and resuspended in 500 µl of PBS and analyzed on FACScan (Beckman-Coulter Inc., CA). The purity of the neutrophil preparation was also confirmed by staining using FITC labelled anti-CD16 mAb or isotype control IgG1 mAb(1/100 dilution). FACS analysis was done with CellQuest Software (Beckman-Coulter Inc). The results are presented as overlaid histograms and as percentage of positive cells.

### Quantitative indirect immunofluorescence test

Quantitative determination of FcεRI α chain cell surface expression in human neutrophils and basophilic cell line (KU812) was performed using QIFIT as recommended by the supplier (DakoCytomation, CA). Two beads were used in this protocol. Setup beads were a mixture of blank and high fluorescence beads that were used to establish the fluorescence window of analysis of flow cytometer. The voltage of the fluorescence detector (PMT) was adjusted to make sure that both negative cells and the two populations of beads were displayed simultaneously on the fluorescence scale. The calibration beads were a mixture of beads with well-known numbers of antibody binding sites per bead; fluorescence data corresponding to each of the five bead peaks were used to construct the calibration curve of mean fluorescence intensity (MFI) against binding capacity (ABC). Briefly, neutrophils and KU812 (1×10^5^ /100 µl) were labelled under saturating conditions with mAbCRA-1 or isotype IgG_2a_ control (10 µg/ml). After washing, cells and setup beads and calibration beads were stained, in parallel, with affinity purified FITC conjugated rat anti-mouse IgG F(ab)′_2_ in saturating amounts (1∶200). Cells were analyzed on the flow cytometer and ABC was calculated based on the equation of the calibration curve [38].

### FISH

FcεRI α chain probe was generated by RT-PCR and subcloned into pBluescript vector (Stratagene, Mississauga, Ontario) [19]. Vector was linearized with the appropriate enzymes and transcribed with digoxigenin UTP (Roche, Mississauga, Ontario) to generate sense and anti-sense riboprobes as we described previously [19]. For ISH procedures, slides were washed, prehybridized with hybridization buffer (50% Formamide, 5X SSC, 100 µg/ml Heparin, 1X- Denhardt's solution, 0.1% Tween 20, 0.1% CHAPS, 5mM EDTA and 0.3mg/ml yeast tRNA), followed by adding digoxigenin labeled anti-sense probe (1 µg/ml) overnight at 42°C. Digoxigenin labelled sense probe was used as negative control. The slides were washed three times with 0.2X SSC for 30 min at 65°C and PBT (0.1% Triton X 100, 2 µg/ml BSA, PBS) for 20 min at room temperature. After endogenous peroxide activity blocking, the slides were washed with TNT (0.1M Tris HCl pH7.5, 0.15M NaCl, 0.05% Tween 20) for 5min each at RT, incubated with mouse monoclonal anti-digoxigenin (1∶100) for 30min at RT, followed by tyramide signal amplification (TSA) step using HRP conjugated goat anti-mouse IgG (H+L) and Alexa Fluor 488 tyramide as described by the manufacturer (Invitrogen, Carlsabd, CA). TSA is an ultrasensitive molecular morphology detection technique that utilizes the catalytic activity of horseradish peroxidase to generate robust labeling of target nucleic acid sequence or protein in situ in tissue sections or on cells in suspension [39]. Counterstaining was performed with propidium iodide. After washing with PBS, the slides were mounted with ProLong® antifade**.** Images were acquired under oil immersion at 100x magnification by confocal laser scanning microscopy (Olympus IX70 inverted microscope coupled to FluoVIEW confocal laser scanning system with a Cooke Sensicam (Olympus America Inc., Melville, NY). The value of ISH represents the percentage of cells that had a clear signal compared to background signal obtained with the sense probe. This was determined by counting the positive and negative cells, blindfolded for subjects and is the average of three different counts by two different individuals. The background signal represents the number of green dots per cell obtained with sense riboprobe. Cells hybridized with sense riboprobe exhibited no or 1 to 3 green dots as background signal. Cells showing the same number of green dots as obtained with the sense riboprobe are considered negative.

### Confocal laser scanning microscopy and immuno-fluorescence

This was performed by incubating the slides with universal blocking solution (DAKO Diagnostics Canada Inc., Mississauga, ON) for 20 min at RT, followed by overnight incubation at 4°C with mAb-CRA1 or isotype control mAb IgG_2a_ (both at 10 µg/ml) prepared in antibody diluting buffer (DAKO Cytomation, Ca). The slides were washed in TBS and incubated with 1∶100 dilution of FITC conjugated affinity pure rat anti-mouse IgG (H+L). Nuclear counterstaining and image acquisition was performed as described above. FluoVIEW 2.0 software (Olympus America Inc., Melville, NY) was used to acquire and analyze the confocal images.

### RNA isolation and Real time RT-PCR

Total cellular RNA was extracted using Trizol (Invitrogen). Reverse transcription (RT) was performed by using 2 µg of total RNA in a first strand cDNA synthesis reaction with SuperScript reverse transcriptase as recommended by the supplier (Invitrogen). The quantification of mRNA of FcεRI-α chain expressed by neutrophils was performed using *Real-Time RT-PCR* by the Light Cycler ™ . Primer set for FcεRI α chain were: 5′CTCCATTACAAATGCCACAGT3′and 5′CACGCGGAGCTTTTATTACAGTA3′. Primers for house-keeping gene glyceraldhyde-3-phosphate dehydrogenase (GAPDH), and standard controls were developed in our laboratory. The forward and reverse specific primer sequences used, the size of the amplified fragment and the annealing temperature for GAPDH are: 5′-AGCAATGCCTCCTGCACCACCAAC-3′ and 5′-CCGGAGGGGCCATCCACAGTCT-3′, 137 bp, 59°C. DNA standards were prepared from PCR using cDNA from cells stimulated by IL-1β. PCR products were isolated from 0.5% w/v agarose gel using QIAEX II Agarose Gel Extraction kit (Qiagen). The amount of extracted DNA was quantified by spectrophotometry and expressed as copy number. A serial dilution was used to generate each standard curve. PCR was performed by LightCycler-DNA Master SYBR Green I as ready to use reaction mix (Roche). Calculation of the amount of each cDNA species was performed according to manufacturer's standard protocol. Briefly, the amplification of target genes in stimulated cells was calculated by normalizing to the amplification of GADPH gene in the sample.

### Immunoprecipitation and Western blot

For protein extraction, neutrophils were lysed for 30 min at 4°C in NP-40 lysis buffer supplemented with a cocktail of protease inhibitors (2mM sodium orthovanadate, 1mM phenyl-methylsulfonylfluoride, 10 µg/ml leupeptin, 0.15 units/ml approtinin, 1 µg/ml pepstatin A) (Sigma) and centrifuged for 20 min to remove nuclei. Cell lysates from neutrophils or basophilic cell line (KU812, ATCC® # CRL-2099™) used as positive control were sequentially incubated with 2 µg/ml of human IgE (Diateck, Oslo, Norway) for 16 h at 4°C in a rotating mixer, followed by protein G sepharose-coated beads (Amersham-Pharmacia) conjugated with mouse mAb anti-human IgE (Pharmingen) for 2h at 4°C. Immuno-complexes were pelleted by centrifugation and washed six times with the wash buffer (PBS /1% NP40). For immunoblotting, samples were separated on SDS polyacrylamide gel (13%) and electro-transferred onto PVDF membrane (Millipore, Missisauga, Ontario). The membrane was blocked at RT for 2 hrs with 5% Blotto, (Santa Cruz Biotechnology, CA, USA), incubated with mouse anti-FcεRI-α chain mAbCRA-1 (1 µg/ml) at room temperature for 2 h, followed by secondary antibody HRP-goat anti-mouse IgG (H+L) prepared in TBST (1∶5000). The blots were developed by enhanced chemiluminiscence as recommended by the supplier (Amersham Pharmacia,ON ).

### Statistics

Statistical analysis was performed using GraphPad Prism Software Version 3·02 for Windows (GraphPad Software, San Diego, CA, USA). The association between levels of FcεRI expression during and out of allergic season was determined by Wilcoxon signed rank test (paired, non parametric). Association between expression levels in the subgroups and cytokine stimulation effect on FcεRI expression were studied using Mann-Whitney U test (unpaired non parametric).

## Supporting Information

Figure S1 The effect of IgE on FcRI-α chain expression in neutrophils. Basophilic cell line (KU8112) and neutrophils from allergic asthmatics (n = 11) were stimulated or not with IgE (10μg/ml) for 24h. FcRI expression was analyzed by FACS. B-FcRI α chain antigen surface expression in human neutrophils and human basophilic cell line tested with quantitative indirect immunofluorescence test.
(0.58 MB TIF)
